# Novel catenated N_6_ energetic compounds based on substituted 1,2,4-triazoles: synthesis, structures and properties†

**DOI:** 10.1039/c8ra02491j

**Published:** 2018-04-16

**Authors:** Yanan Li, Bin Wang, Pei Chang, Jianjian Hu, Tao Chen, Yinglei Wang, Bozhou Wang

**Affiliations:** State Key Laboratory of Fluorine & Nitrogen Chemicals, Xi'an Modern Chemistry Research Institute Xi'an Shanxi 710065 P. R. China lyn2003080094@126.com wangyl204@163.com

## Abstract

1-Amino-3,5-dinitro-1,2,4-triazole (ADNT) was prepared using an efficient N-amination process. Three novel catenated N_6_ energetic derivatives of ADNT, which contain 1,1′-azobis(3,5-dinitro-1,2,4-triazole) (ABDNT), 1,1′-azobis(3-chloro-5-nitro-1,2,4-triazole) (ABCNT) and 1,1′-azobis(3,5-diazido-1,2,4-triazole) (ABDAT), were synthesized from N-amino oxidative-coupling reactions of ADNT. All compounds were fully characterized by ^1^H and ^13^C nuclear magnetic resonance spectroscopies, infrared spectroscopy, elemental analysis, mass spectrum, as well as differential scanning calorimetry (DSC). The crystal structure of compound ABCNT was confirmed by single-crystal X-ray diffraction showing an extensive conjugated structure. The densities of energetic derivatives ranged from 1.71 to 1.93 g cm^−3^, and all compounds have positive heats of formation in the range of 774.8 to 2150.8 kJ mol^−1^. Based on the measured densities and calculated heats of formation, theoretical performance calculations, including detonation pressures (29.6–42.4 GPa) and detonation velocities (8.22–9.49 km s^−1^) were carried out using the Gaussian 09 program and Kamlet–Jacobs equations, and they compared favorably with those of TNT and RDX. These properties make them potentially competitive as new high energy-density compounds.

## Abstract 2

Novel catenated N_6_ energetic compounds based on substituted 1,2,4-triazoles were synthesized, which can be used as new high energy-density materials.

## Graphical abstract

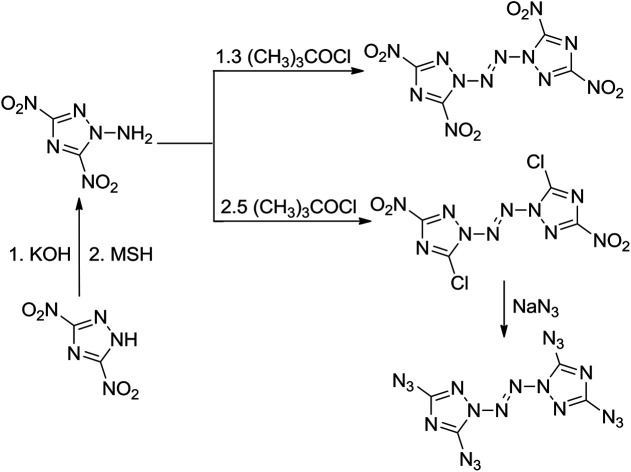

## Introduction

**Scheme 1:**
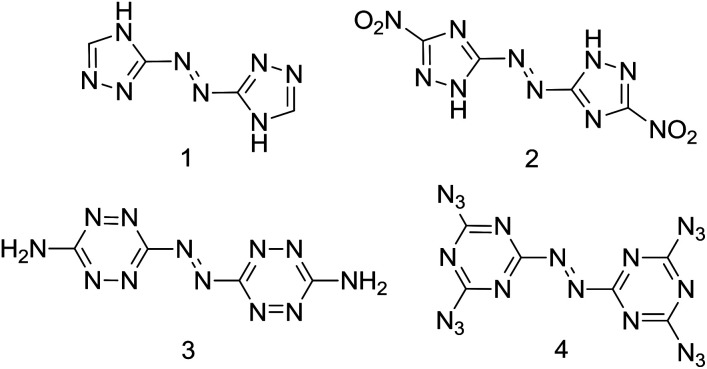
Examples of reported high-nitrogen compounds with C,C′-azo linkage.

Traditional energetic materials including quintessential explosives such as TNT (2,4,6-trinitrotoluene), RDX (1,3,5-trinitro-1,3,5-triazinane) and HMX (1,3,5,7-tetranitro-1,3,5,7-tetrazocane) are based on the oldest strategy in the design of energetic materials: the presence of fuel and oxidizer in the same molecule. New strategies in energetic materials' research focus on nitrogen-rich/high positive heat of formation compounds.^1^ Nitrogen-rich compounds have therefore received increasing attention as promising candidates for high energy-density materials (HEDM) which might be used as propellants, explosives or especially as gas generators,^2^ and the generation of nitrogen gas as a final product of nitrogen-rich compounds is highly favored for the enhancement of energy and avoiding environmental pollution.^3^ Nitrogen-rich compounds based on C/N heteroaromatic rings with high nitrogen content like triazole, tetrazole, triazine and tetrazine are at the forefront of high energy research.^4^ Recently, the combination of an azo group with nitrogen-rich heteroaromatic rings has been extensively studied because the azo linkage not only desensitizes but also dramatically increases the heats of formation of high-nitrogen compounds such as 3,3′-azobis(1,2,4-triazole) (1), 5,5′-dinitro-3,3′-azo-1*H*-1,2,4-triazole (2), 3,3′-azobis-(6-amino-1,2,4,5-tetrazine) (3), 4,4′,6,6′-tetra(azido)azo-1,3,5-triazine (4) and are four representative compounds of this kind (Scheme 1),^5^ where the two heteroaromatic rings are connected by a C–N=N–C linkage. However, if the azo group is attached to the nitrogen of the heteroaromatic rings to create a rather long chain of catenated nitrogens (N–N=N–N linkage), such a polynitrogen structure could result in unique properties and features of these energetic compounds.

**Scheme 2:**
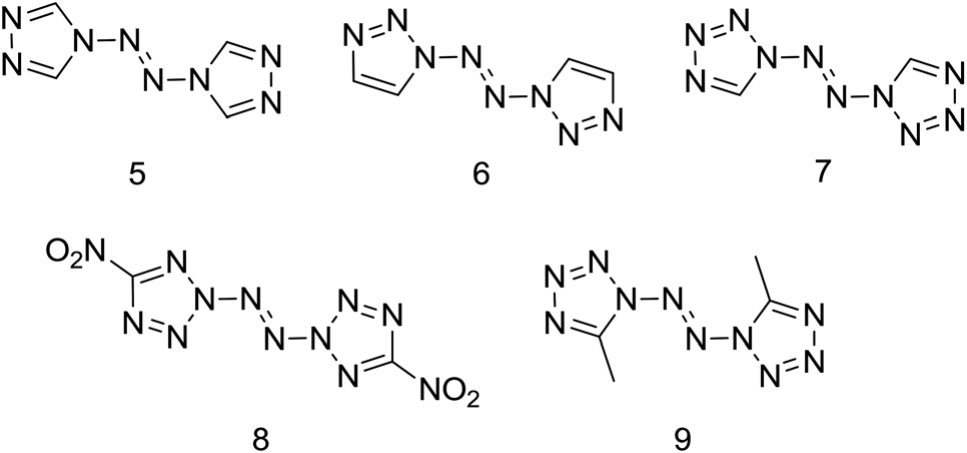
Examples of reported high-nitrogen compounds with *N*,*N*′-azo linkage.

In the pursuit of novel nitrogen-rich energetic materials, some catenated nitrogen-atom compounds containing the N–N=N–N linkage have been synthesized over the past years, such as 4,4′-azo-1,2,4-triazole (5),^5*a*,6^ 1,1′-azobis-1,2,3-triazole (6), 1,1′-azobis(tetrazole) (7), 2,2′-azobis(5-nitrotetrazole) (8) and 1,1′-azobis(5-methyltetrazole) (9) (Scheme 2).^7^ Unfortunately, most of the above compounds don't own excellent detonation performance (such as compounds 5, 6, 9) or thermal unstable (such as compounds 7, 8, 9).

The N-amination of electron-rich systems, such as imidazoles, pyrazoles, triazoles and tetrazoles, have been reported for several researchers,^7*c*,8^ using the commercially available hydroxylamine-*O*-sulfonic acid (HOSA) as N-amination agent. However, HOSA aminations unfortunately do not apply to electron-poor systems. The addition of an amino group to energetic compounds could improve the stability, for example amino–nitro compounds such as diaminodinitroethylene (FOX-7) or 2,6-diamino-3,5-dinitropyrazine-1-oxide (LLM-105) are low-sensitivity and high-performance explosives that are the standard for insensitive explosives.^7*c*,9^ N-Amino compounds have the further advantage that their heats of formation and other explosive performance increase as a result of the additional N–N bond. Furthermore, new potential nitrogen ligands for high energy-density materials can be designed and synthesized by the N–NH_2_ group of N-amino compounds.

**Scheme 3:**
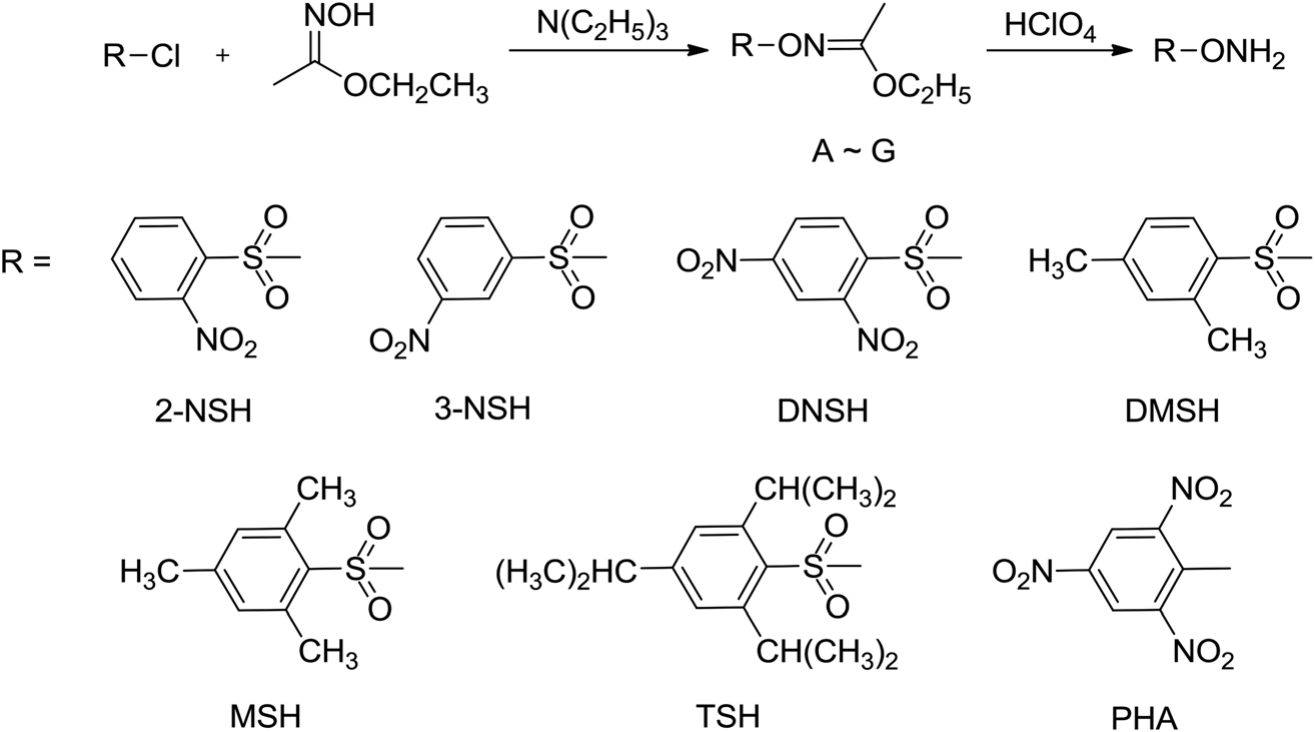
Synthesis routes of N-amination agents.

**Scheme 4:**
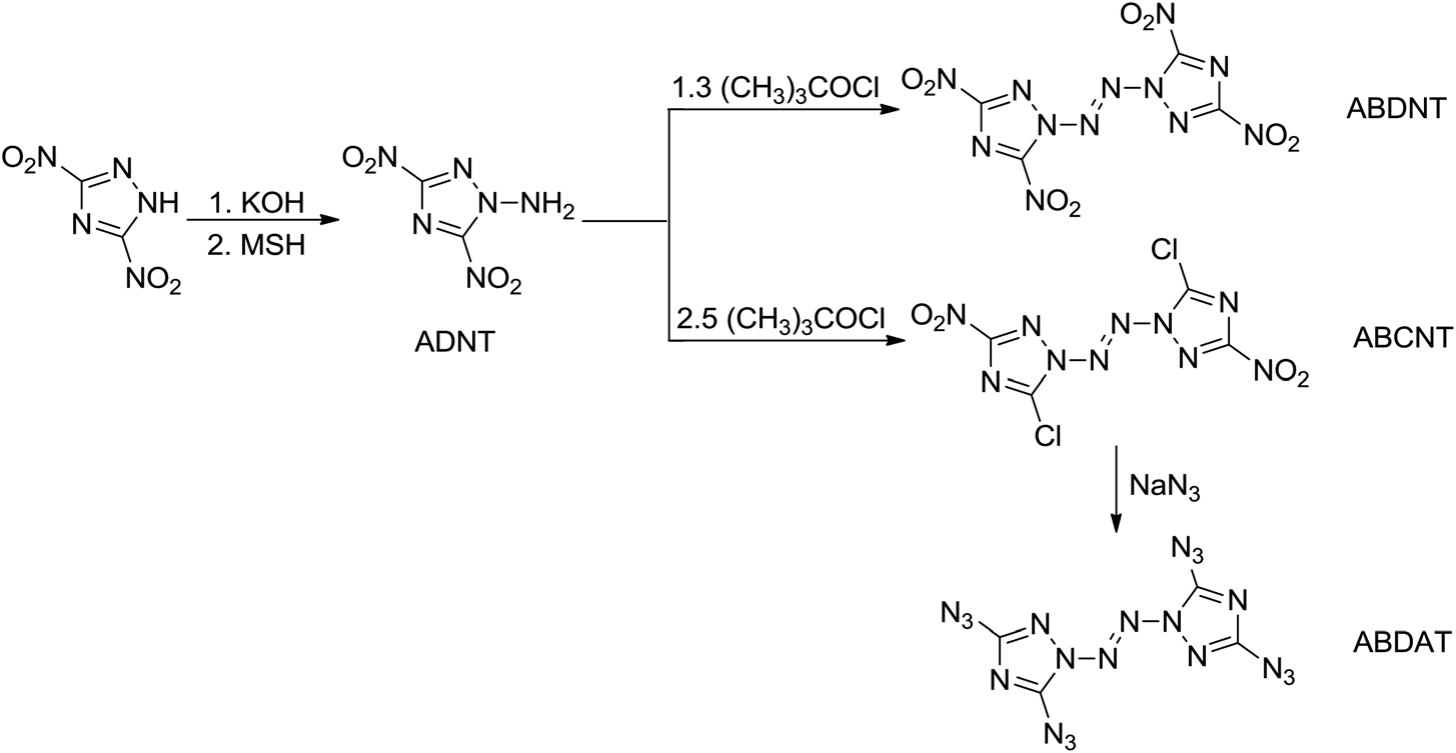
The synthetic pathways of energetic derivatives based on ADNT.

*O*-Tosylhydroxylamine (THA)^10^ is a powerful amination agent well known to aminate electron-poor systems, but THA is unfortunately not stable in storage under room temperature condition, so before each reaction it was firstly prepared. In this paper, several new N-amination agents were synthesized (Scheme 3), and the thermal stability were investigated by DSC method. Among of them, 2,4,6-trimethylbenzenesulfonyl hydroxylamine (MSH), 2,4,6-triisopropylbenzenesulfonyl hydroxylamine (TSH) and 2,4,6-trinitrophenyl hydroxylamine (PHA) are stable in storage under room temperature. We have used MSH to aminate the electron-poor 3,5-dinitro-1,2,4-triazolate anion yielding 1-amino-3,5-dinitro-1,2,4-triazole (ADNT). Three energetic derivatives of ADNT have also been prepared: the azo coupling product of ADNT, 1,1′-azobis(3,5-dinitro-1,2,4-triazole) (ABDNT); the azo coupling and chlorination product of ADNT, 1,1′-azobis(3-chloro-5-nitro-1,2,4-triazole) (ABCNT), and the azidation product of ABCNT, 1,1′-azobis(3,5-diazido-1,2,4-triazole) (ABDAT) (Scheme 4). All the compounds were characterized by ^1^H and ^13^C nuclear magnetic resonance spectroscopies, infrared spectroscopy, elemental analysis, mass spectrum and differential scanning calorimetry (DSC). The single crystal of ABCNT was determined by X-ray diffraction. Computational calculations confirming the high energetic performance of all derivatives were also performed.

## Results and discussion

### Synthesis of N-amination agents

The commercially hydroxylamine-*O*-sulfonic acid (HOSA) as N-amination agent could only be used to the N-amination reaction of electron-rich systems, and the yields of N-amination products were always lower. *O*-Tosylhydroxylamine (THA) is an efficient N-amination agent that could aminate electron-poor systems and have better yields of targets, but THA is not stable in storage under room temperature condition.^7*c*,8,10^ In our studies, seven new N-amination agents, such as 2-nitrobenzenesulfonyl hydroxylamine (2-NSH), 3-nitrobenzenesulfonyl hydroxylamine (3-NSH), 2,4-dinitrobenzenesulfonyl hydroxylamine (DNSH), 2,4-dimethylbenzenesulfonyl hydroxylamine (DMSH), 2,4,6-trimethylbenzenesulfonyl hydroxylamine (MSH), 2,4,6-triisopropylbenzenesulfonyl hydroxylamine (TSH) and 2,4,6-trinitrophenyl hydroxylamine (PHA), were synthesized using *O*-substituted benzenesulfonyl chloride and ethyl acetohydroxamate as starting materials by the reactions of condensation and hydrolysis. The compounds 2-NSH, 3-NSH, DNSH, and DMSH easily decomposed in the solid state at room temperature more than half an hour, but they was stable in the solution at lower temperature for several weeks. According to the results of DSC experiments, the compounds MSH, TSH, and PHA could be stable in storage under ambient condition. The thermal decomposition temperatures were 79.3 °C, 107.7 °C, and 94.1 °C, respectively.

### Synthesis of ADNT and its energetic derivatives

Various *N*,*N*′-diazo-bridged compounds of single-ring azole-based compounds, such as substituted pyrazole, imidazole, 1,2,3-triazole and tetrazole, have been synthesized by several research groups.^5*a*,6,7,8*a*,8*b*^ N-amino azo-coupling products of substituted 1,2,4-triazole, however, have not been fully explored and fully characterized.^8*b*^ 3,5-dinitro-1,2,4-triazole (DNT) reacted with potassium hydroxide ethanol solution to produce potassium 3,5-dinitro-1,2,4-triazolate (KDNT), followed by amination reaction with the MSH as the N-amination agent to yield N-amino compound 1-amino-3,5-dinitro-1,2,4-triazole (ADNT). Treatment of an acetonitrile solution of ADNT with *tert*-butyl hypochlorite (*t*-BuOCl) led to obtain two different compounds. When the molar ratio of *t*-BuOCl and ADNT was 1.3 : 1, the azo coupling product was 1,1′-azobis(3,5-dinitro-1,2,4-triazole) (ABDNT). When the molar ratio of *t*-BuOCl and ADNT was more than 2.5 : 1, the azo coupling product was 1,1′-azobis(3-chloro-5-nitro-1,2,4-triazole) (ABCNT); the chlorine atoms and nitro groups in ABCNT are certainly activated and are reacted with nucleophiles. The chlorine atoms and nitro groups in compound ABCNT were substituted with azide groups by reaction with sodium azide to produce 1,1′-azobis(3,5-diazido-1,2,4-triazole) (ABDAT). The synthetic pathways to ADNT and its all energetic derivatives are depicted in Scheme 4. The structures of all compounds were fully characterized by IR spectroscopy, DSC, ^1^H and ^13^C NMR spectroscopies and elemental analysis. The data are listed in the Experimental section.

### Sensitivities

To evaluate the stabilities of these *N*,*N*′-diazo-bridged energetic compounds, we studied the impact and friction sensitivities. The sensitivities of all explosives were determined experimentally according to standard BAM methods.^11^ As can be seen in Table 1, The impact sensitivities of these energetic derivatives range from 6 J to 20 J. The value of friction sensitivities are from 80 N to 300 N. The compounds ABDNT and ABCNT are considerably less sensitive toward impact and friction sensitivities than RDX and HMX (RDX: IS = 7.4 J, FS = 120 N; HMX: IS = 7.4 J, FS = 112 N), which suggest that they can serve as promising candidates for safe energetic materials. All the novel catenated N_6_ energetic derivatives based on ADNT are less sensitive than the reported high-nitrogen compounds with *N*,*N*′-diazo-bridged compounds (N_8_ and N_10_ compounds).

**Table 1:** Physiochemical and detonation properties of novel catenated N_6_ energetic derivatives based on ADNT

Compound	ABDNT(N_6_)	ABCNT(N_6_)	ABDAT(N_6_)	6(N_8_)	7(N_10_)	8(N_10_)	9(N_10_)	TNT	RDX	HMX
*T*_dec_ a (°C)	262.4	—	168.8	187.5	80	50	112.5	295.0	239.2	287.0
OB^b^ (%)	0	−25.4	−39.0	−97.6	−48.2	0	−90.7	−74.0	−21.6	−21.6
*N* c (%)	48.8	43.4	85.3	68.3	84.3	65.6	72.1	18.5	37.8	37.8
*ρ* d (g cm^−3^)	1.93	1.92^k^	1.71	1.62	1.77	1.80	1.48	1.65	1.82	1.90
Δ*H*_f_^e^ (kJ mol^−1^)	973.9	774.8	2150.8	962.3	1030.0	1153.0	986.1	−115.0	92.6	104.8
*D* f (km s^−1^)	9.49	8.83	8.22	7.76	9.18	9.18	7.32	6.88	8.71	9.10
*P* g (GPa)	42.4	35.8	29.6	25.2	36.1	39.0	21.0	19.5	33.7	39.6
*Q* h (kJ kg^−1^)	7476	6283	6629	—	—	6931	5078	4222	5355	5695
IS^i^ (J)	10	20	6	4.1	≪1	⋘1	—	15	7.4	7.4
FS^j^ (N)	160	300	80	—	≪5	⋘5	—	353	120	112

a Thermal decomposition temperature.

b Nitrogen content.

c Oxygen balance (based on CO_2_) for C_*a*_H_*b*_O_*c*_N_*d*_, 1600(c−2a−b/2)/MW, MW = molecular weight.

d Density measured by gas pycnometer.

e Heat of formation.

f Detonation velocity.

g Detonation pressure.

h Heat of detonation.

i Impact sensitivity.

j Friction sensitivity.

k Single crystal density (296 K).

### Physiochemical and detonation properties

The thermal stabilities of compounds ABDNT and ABDAT were determined by differential scanning calorimetric (DSC) measurements at a heating rate of 5 °C min^−1^. The thermal decomposition peak temperatures of ABDNT and ABDAT were 262.4 °C and 168.8 °C (seen in Table 1), respectively, compared with that of traditional energetic compound RDX (239.2 °C). Because of the existence of the four azide groups in one molecular structure, compound ABDAT has lower decomposition temperature (168.8 °C). However, the decomposition peak temperatures of ABDNT and ABDAT are much higher than those of hexazene (N_6_) ligand (140 °C) and N_5_^+^ (70 °C).^12^

Densities of these energetic compounds which are in the range of 1.71 to 1.93 g cm^−3^ were determined using gas pycnometer or single-crystal X-ray diffraction. It is noteworthy that the densities of compounds ABDNT and ABCNT fall in the range designated for new HEDMs (1.8–2.0 g cm^−3^),^13^ which are higher than RDX (1.82 g cm^−3^). Moreover, the high densities of ABDNT (1.93 g cm^−3^) and ABCNT (1.92 g cm^−3^) are even comparable with that of HMX (1.90 g cm^−3^).

Heats of formation are one of the important characteristics for energetic compounds and are directly related to the number of nitrogen–nitrogen bonds in molecular structures. The standard enthalpies of formation for these derivatives were calculated by using the Gaussian 09 (Revision A. 02)^14^ suite of programs and atomization method based on CBS-4M enthalpies.^15^ All of the optimized structures were characterized to be true local energy minima on the potential-energy surface without imaginary frequencies. As shown in Table 1, the solid phase heats of formation for *N*,*N*′-diazo-bridged energetic derivatives based on ADNT are highly endothermic compounds. All of these compounds exhibit higher positive heats of formation ranging between 774.8 and 2150.8 kJ mol^−3^, which are compared with those of RDX (92.6 kJ mol^−3^), HMX (104.8 kJ mol^−3^), and other reported catenated nitrogen-atom compounds (N_8_ and N_10_ structures in Table 1). Especially, compound ABDAT exhibits the highest heat of formation (2150.8 kJ mol^−1^) among of them because of four azido-functionalization groups in the same molecular structure.

Based on the calculated values of heats of formation and the experimental values for the densities of these energetic derivatives, the detonation velocities (*D*) and detonation pressures (*P*) were calculated using the Kamlet–Jacobs equations.^16^ The detonation velocities (*D*) lie between 8.22 and 9.49 km s^−1^ (compared with TNT 6.88 km s^−1^, and RDX 8.71 km s^−1^). Detonation pressures of these compounds lie in the range between 29.6 to 42.4 GPa (compared with TNT 19.5 GPa, and RDX 33.7 GPa). Compound ABDNT has the highest detonation velocity (9.49 km s^−1^) and detonation pressure (42.4 GPa), which are also higher than other reported *N*,*N*′-diazo-bridged energetic compounds (N_8_ and N_10_ structures). Although not all these energetic derivatives perform better than RDX by calculations, they can probably find use in certain applications in civilian use or in military applications.

### X-Ray crystallography

**Fig. 1:**
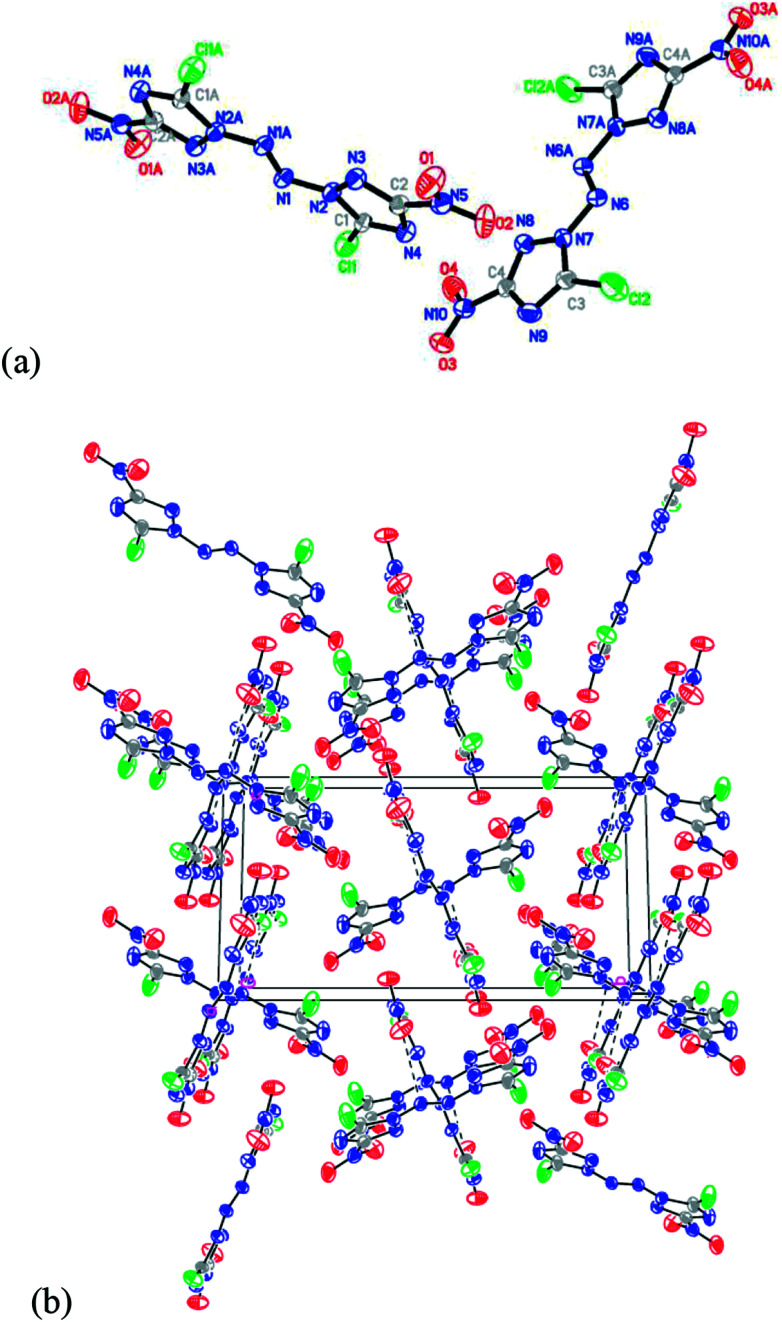
(a) X-ray structure of ABCNT with thermal ellipsoids at 50% probability. (b) Packing diagram of ABCNT viewed down the *a* axis.

A single crystal of compound ABCNT suitable for crystal structure analysis was obtained by slow evaporation from acetonitrile solution of ABCNT at room temperature. The crystal data and structure refinement details of ABCNT are given in Table 2. The crystal structure of ABCNT adopts a planar structure with two almost planar 3-chloro-5-nitro-1,2,4-triazole rings, a planar N_6_ chain and an *E* configuration about the azo bond (Fig. 1). The azo bond adopts a stable *E* configuration due to lower active energy than the *Z* configuration. The bond length between the N-atoms of the azo group (N1=N1A) is 1.240 Å, shorter than that of compound 5 with the N_4_ structure (1.249 Å), compound 6 with the N_8_ structure (1.250 Å) and compound 9 with the N_10_ structure (1.243 Å), which indicates a stronger delocalization of the nitro π_3_ (ref. 4) conjugated bond and azo π-bond along the molecular structure within compound 6. But longer than that of N_4_H_4_ (2-tetrazene)^17^ with the N_4_ structure (1.205 Å) and compound 7 with the N_10_ structure (1.178 Å), respectively. This is probably due to the electron-withdrawing property of the nitro groups.

**Table 2:** Crystal data and structure refinement details of ABCNT

Compounds	ABCNT
Empirical formula	C_4_N_10_O_4_Cl_2_
Formula weight	323.04
CCDC number	1826501
*T* (K)	296(2)
*λ* (Å)	0.71073
Crystal system	Monoclinic
Space group	*P*2_1_/*c*
Unit cell dimensions (Å, °)	*a* = 8.844(5), *α* = 90
	*b* = 15.589(9), *β* = 105.495(11)
	*c* = 8.401(5), *γ* = 90
*V* (Å^3^)	1116.2(12)
*Z*	4
*D*_c_ (g cm^−3^)	1.922
Absorption coefficient (mm^−1^)	0.619
*F* (000)	640
Goodness-of-fit on *F*^2^	1.001
Final *R* indices (*I* > 2*σ*(*I*))	*R*_1_ = 0.0536, w*R*_2_ = 0.1352
Largest diff. peak and hole (e Å^−3^)	0.302 and −0.378

## Experimental

### Caution

Although we experienced no problems during the synthesis of the target compounds, standard safety precautions (leather gloves, face shield and ear plugs) should be used when handling these energetic materials.

### General methods

All chemical reagents and solvents (analytical grade) were used as supplied unless otherwise stated. Infrared spectra were obtained from KBr pellets on a Nicolet NEXUS870 Infrared spectrometer in the range of 4000–400 cm^−1^. ^1^H NMR and ^13^C NMR were obtained in DMSO-*d*_6_ on a Bruker AV500 NMR spectrometer. Elemental analyses (C, H and N) were performed on a VARI-El-3 elementary analysis instrument. Mass spectra were acquired using a GCMS-QP 2010 Micromass UK spectrometer. The DSC experiment was performed using a DSC-Q 200 apparatus (TA, USA) under a nitrogen atmosphere at a flow rate of 50 mL min^−1^. About 0.5–1.0 mg of the sample was sealed in aluminium pans for DSC. For all energetic materials, the impact sensitivity were determined with a ZBL-B impact sensitivity instrument, the friction sensitivity were determined with a FSKM 10 friction sensitivity instrument. Energetic properties have been calculated with Gaussian 09 program^14^ and Kamlet–Jacobs equations^16^ using the calculated solid state heat of formation and experimental value of density. These were computed by the atomization method as described in published papers.

### X-ray crystallography

Single crystal suitable of compound ABCNT for X-ray measurement was obtained by slow evaporation of acetonitrile solution of ABCNT at room temperature. The crystal structure of ABCNT was determined by a Bruker SMART APEXII CCD X-ray diffractometer and the SHELXTL crystallographic software package. A single crystal was mounted on a Bruker SMART APEXII CCD X-ray diffractometer equipped with graphite-monochromatized Mo-Kα radiation (0.71073 Å). Data were collected by the ω scan technique. The structure was solved by direct methods using SHELXS-97 program^18^ and refined against *F*^2^ by full-matrix least-squares using SHELXL-97 program.^19^

### Theoretical study

All the quantum computations were performed using the Gaussian 09 (Revision A. 02) suite of programs.^14^ The optimized structures were characterized to be true local energy minima on the potential-energy surface without imaginary frequencies. The room-temperature gas-phase enthalpies of all energetic compounds were obtained by the atomization method based on CBS-4M calculated electronic enthalpies using NIST^20^ values as standardized values for the standard heats of formation (Δ*H*_f_°). Often the standard state of the materials of interest corresponds to the solid phase. Thus, the solid state enthalpies of formation can be determined using the gas-phase enthalpy of formation and enthalpy of sublimation phase transition according to the Hess' law of constant heat summation as the eqn (1).^21^

1 Δ*H* (solid) = Δ*H* (gas) − Δ*H* (sublimation)

Based on the electrostatic potential of a molecule by quantum mechanical prediction, the heat of sublimation can be represented as the eqn (2).^22^

2 Δ*H* (sublimation) = *a*(SA)^2^ + *b*(*σ*_Tot_^2^*ν*)^1/2^ + *c*

where, (SA) is the molecular surface area for this structure, *σ*_Tot_^2^ is described as an indicator of the variability of the electrostatic potential on the molecular surface, *ν* is interpreted as showing the degree of balance between the positive and negative potentials on the molecular surface and *a*, *b*, and *c* are the fitting parameters. We further followed the approach of Politzer to predict the heats of sublimation of energetic materials, and then combined these with eqn (1) and (2) to predict the solid enthalpies of formation.

The empirical Kamlet–Jacobs (K–J) equations^16^ widely employed to evaluate the energy performance of energetic compounds were used to estimate the detonation velocity (*D*) and detonation pressure (*P*) of all target compounds. Empirical Kamlet–Jacobs (K–J) equations can be shown in eqn (3)–(5).

3 *P* = 1.558*ρ*^2^*Φ*

4 *D* = 1.01*Φ*^1/2^(1.011 + 1.312*ρ*)

5 *Φ* = 0.4889*N*(*MQ*)^1/2^

where, *D* is the predicted detonation velocity (m s^−1^), *P* is the predicted detonation pressure (GPa), *ρ* is the density of explosives (g cm^−3^), *N* is the moles of detonation gases per gram explosive, *M* is the average molecular weight of these gases, and *Q* is the chemical energy of detonation (kJ g^−1^). The densities and the calculated heats of formation were used to compute the *D* and *P* values.

### General procedure for *O*-substituted benzenesulfonyl ethyl acetohydroxamate (A–G)

Ethyl acetohydroxamate (5.15 g, 50.0 mmol) was dissolved in *N*,*N*-dimethylformamide (25.0 mL) at room temperature, then triethylamine (5.05 g, 50.0 mmol) was added dropwise. The mixture was kept at 20–25 °C for another 10 minutes and was then cooled to 0–5 °C. *O*-substituted benzenesulfonyl chloride (50.0 mmol) was slowly added to the above cooled mixture while maintaining the reaction temperature below 5 °C. After complete addition, the reaction mixture was stirred for 1 h at 10 °C. When the reaction solution was poured into ice-water, a solid precipitated. The solid was filtered off, washed with cold water, and dried in air to give product *O*-substituted benzenesulfonyl ethyl acetohydroxamate.

#### Compound A

White solid, with a yield of 83.3%. ^1^H NMR ([D_6_]DMSO, 500 MHz, 25 °C, TMS): *δ* = 1.15 (t, 3H), 2.09 (s, 3H), 3.92 (t, 2H), 7.95 (t, 1H), 8.05 (t, 1H), 8.12 (d, 1H), 8.17 (d, 1H) ppm. ^13^C NMR ([D_6_]DMSO, 125 MHz, 25 °C): *δ* = 14.19, 15.37, 64.54, 125.34, 126.72, 132.68, 133.19, 136.91, 148.48, 171.65 ppm. IR (KBr pellet): *ν* = 3101, 2992, 2946, 1620, 1538, 1442, 1382, 1323, 1195, 1127, 1058, 956, 886, 826 cm^−1^. Anal. calcd for C_10_H_12_N_2_O_6_S: C 41.66, H 4.20, N 9.72%; found C 41.79, H 4.12, N 9.82%.

#### Compound B

White solid, with a yield of 75.0%. ^1^H NMR ([D_6_]DMSO, 500 MHz, 25 °C, TMS): *δ* = 1.18 (t, 3H), 2.03 (s, 3H), 3.96 (t, 2H), 8.00 (t, 1H), 8.39 (d, 1H), 8.58 (s, 1H), 8.63 (d, 1H) ppm. ^13^C NMR ([D_6_]DMSO, 125 MHz, 25 °C): *δ* = 14.23, 15.34, 64.47, 123.77, 129.64, 132.11, 134.85, 136.29, 148.36, 171.40 ppm. IR (KBr pellet): *ν* = 3104, 2993, 2953, 1632, 1534, 1379, 1353, 1197, 1053 cm^−1^. Anal. calcd for C_10_H_12_N_2_O_6_S: C 41.66, H 4.20, N 9.72%; found C 41.58, H 4.32, N 9.61%.

#### Compound C

Light-yellow solid, with a yield of 70.9%. ^1^H NMR ([D_6_]DMSO, 500 MHz, 25 °C, TMS): *δ* = 1.18 (t, 3H), 2.10 (s, 3H), 3.93 (t, 2H), 8.42 (d, 1H), 8.66 (d, 1H), 9.02 (d, 1H) ppm. ^13^C NMR ([D_6_]DMSO, 125 MHz, 25 °C): *δ* = 14.26, 15.48, 64.76, 120.99, 127.63, 131.36, 134.57, 148.52, 151.66, 172.26 ppm. IR (KBr pellet): *ν* = 3111, 3092, 2999, 1630, 1559, 1539, 1350, 1326, 1192, 1051, 959 cm^−1^. Anal. calcd for C_10_H_11_N_3_O_8_S: C 36.04, H 3.33, N 12.61%; found C 36.24, H 3.24, N 12.71%.

#### Compound D

Yellowish solid, with a yield of 70.9%. ^1^H NMR ([D_6_]DMSO, 500 MHz, 25 °C, TMS): *δ* = 1.20 (t, 3H), 2.05 (s, 3H), 2.56 (s, 3H), 2.90 (s, 3H), 3.93 (t, 2H), 7.2 (d, 1H), 7.8 (d, 1H), 8.12 (s, 1H) ppm. ^13^C NMR ([D_6_]DMSO, 125 MHz, 25 °C): *δ* = 13.88, 14.76, 20.98, 22.64, 63.49, 129.13, 130.53, 133.24, 135.61, 141.67, 142.36, 170.31 ppm. IR (KBr pellet): *ν* = 2993, 2952, 1641, 1611, 1452, 1361, 1320, 1191, 971 cm^−1^. Anal. calcd for C_12_H_17_NO_4_S: C 53.12, H 6.32, N 5.16%; found C 53.23, H 6.23, N 5.27%.

#### Compound E

White solid, with a yield of 95.6%. ^1^H NMR ([D_6_]DMSO, 500 MHz, 25 °C, TMS): *δ* = 1.19 (t, 3H), 2.04 (s, 3H), 2.31 (s, 3H), 2.65 (s, 6H), 3.91 (t, 2H), 6.97 (s, 2H) ppm. ^13^C NMR ([D_6_]DMSO, 125 MHz, 25 °C): *δ* = 13.98, 14.87, 21.07, 22.83, 63.61, 130.39, 131.52, 140.71, 143.32, 169.24 ppm. IR (KBr pellet): *ν* = 2981, 2941, 1636, 1605, 1454, 1365, 1316, 1180, 1054, 962, 851 cm^−1^. Anal. calcd for C_13_H_19_NO_4_S: C 54.72, H 6.71, N 4.91%; found C 54.63, H 6.82, N 4.98%.

#### Compound F

White solid, with a yield of 91.1%. ^1^H NMR ([D_6_]DMSO, 500 MHz, 25 °C, TMS): *δ* = 1.15 (m, 24H), 2.04 (s, 3H), 3.88 (t, 2H), 7.31 (s, 2H) ppm. ^13^C NMR ([D_6_]DMSO, 125 MHz, 25 °C): *δ* = 14.09, 15.12, 23.76, 24.29, 24.72, 25.27, 28.52, 29.70, 33.91, 64.00, 121.87, 124.20, 129.06, 142.08, 147.30, 147.81, 151.28, 154.47, 169.59 ppm. IR (KBr pellet): *ν* = 2981, 2941, 1636, 1605, 1454, 1365, 1316, 1180, 1054, 962, 851 cm^−1^. Anal. calcd for C_19_H_31_NO_4_S: C 61.76, H 8.46, N 3.79%; found C 61.87, H 8.35, N 3.86%.

#### Compound G

Light-pink solid, with a yield of 85.2%. ^1^H NMR ([D_6_]DMSO, 500 MHz, 25 °C, TMS): *δ* = 1.35 (t, 3H), 2.23 (s, 3H), 3.98 (t, 2H), 8.82 (s, 2H) ppm. ^13^C NMR ([D_6_]DMSO, 125 MHz, 25 °C): *δ* = 14.14, 15.03, 64.34, 123.71, 139.87, 141.32, 150.51, 170.26 ppm. IR (KBr pellet): *ν* = 3092, 2989, 2906, 1542, 1357, 1633 cm^−1^. Anal. calcd for C_10_H_10_N_4_O_8_: C 38.23, H 3.21, N 17.83%; found C 38.33, H 3.18, N 17.78%.

### General procedure for synthesis of N-amination agents

*O*-Substituted benzenesulfonyl ethyl acetohydroxamate (5.0 g) was dissolved in 1,4-dioxane (20.0 mL) at room temperature and was then cooled to 0–5 °C. Then perchloric acid (70%∼72%, 8.0 mL) was slowly added dropwise to the above cooled mixture at 0–5 °C. After complete addition, the reaction mixture was stirred for 0.5–1 h at 5–10 °C. When the reaction solution was poured into ice-water, a solid precipitated. The solid was filtered off, washed with cold water, and dried in air to give target.

#### Compound 2-NSH

Light-yellow solid, with a yield of 74.2%. ^1^H NMR ([D_6_]DMSO, 500 MHz, 25 °C, TMS): *δ* = 6.41 (s, 2H), 6.96 (d, 1H), 7.10 (d, 1H), 7.32 (t, 1H), 7.51 (d, 1H) ppm. ^13^C NMR ([D_6_]DMSO, 125 MHz, 25 °C): *δ* = 114.31, 121.60, 124.99, 130.69, 141.34, 150.10 ppm. IR (KBr pellet): *ν* = 3423, 3092, 3016, 1554, 1510, 1459, 1374, 1274, 1243, 1130 cm^−1^. Anal. calcd for C_6_H_6_N_2_O_5_S: 33.03, H 2.77, N 12.84%; found C 33.20, H 2.84, N 12.92%.

#### Compound 3-NSH

Light-yellow solid, with a yield of 60.8%. ^1^H NMR ([D_6_]DMSO, 500 MHz, 25 °C, TMS): *δ* = 6.18 (s, 2H), 7.68 (t, 1H), 8.04 (d, 1H), 8.21 (d, 1H), 8.36 (s, 1H) ppm. ^13^C NMR ([D_6_]DMSO, 125 MHz, 25 °C): *δ* = 120.53, 123.92, 130.30, 132.43, 147.70, 150.31 ppm. IR (KBr pellet): *ν* = 3396, 3244, 3097, 1609, 1536, 1350, 1239, 1193, 1043 cm^−1^. Anal. calcd for C_6_H_6_N_2_O_5_S: C 33.03, H 2.77, N 12.84%; found C 33.15, H 2.67, N 12.75%.

#### Compound DNSH

Off-white solid, with a yield of 81.0%. ^1^H NMR ([D_6_]DMSO, 500 MHz, 25 °C, TMS): *δ* = 3.94 (s, 2H), 8.42 (d, 1H), 8.67 (d, 1H), 9.03 (d, 1H) ppm. ^13^C NMR ([D_6_]DMSO, 125 MHz, 25 °C): *δ* = 120.98, 127.61, 131.38, 134.58, 148.52, 151.65 ppm. IR (KBr pellet): *ν* = 3412, 3273, 3102, 1612, 1539, 1358, 1241, 1045, 884 cm^−1^. Anal. calcd for C_6_H_5_N_3_O_7_S: C 27.38, H 1.91, N 15.97%; found C 27.51, H 1.83, N 16.04%.

#### Compound DMSH

White solid, with a yield of 70.3%. ^1^H NMR ([D_6_]DMSO, 500 MHz, 25 °C, TMS): *δ* = 2.17 (s, 3H), 2.53 (s, 3H), 6.76 (s, 2H), 7.32 (d, 1H), 7.76 (d, 1H), 8.03 (s, 1H) ppm. ^13^C NMR ([D_6_]DMSO, 125 MHz, 25 °C): *δ* = 21.07, 23.54, 129.91, 130.43, 134.52, 137.87, 142.82, 144.79 ppm. IR (KBr pellet): *ν* = 3339, 3268, 3036, 2985, 2947, 781, 688 cm^−1^. Anal. calcd for C_8_H_11_NO_3_S: C 47.75, H 5.51, N 6.96%; found C 47.86, H 5.42, N 7.05%.

#### Compound MSH

White solid, with a yield of 74.3%. ^1^H NMR ([D_6_]DMSO, 500 MHz, 25 °C, TMS): *δ* = 2.18 (s, 3H), 2.51 (s, 6H), 6.77 (s, 2H), 9.24 (s, 2H) ppm. ^13^C NMR ([D_6_]DMSO, 125 MHz, 25 °C): *δ* = 20.76, 23.18, 130.45, 136.42, 137.14, 142.63 ppm. IR (KBr pellet): *ν* = 3340, 3270, 3035, 2987, 2945, 779, 689 cm^−1^. Anal. calcd for C_9_H_13_NO_3_S: C 50.21, H 6.09, N 6.51%; found C 50.33, H 6.15, N 6.44%. MS (*m*/*z*): 214.10 [M − H]^−^.

#### Compound TSH

White solid, with a yield of 91.4%. ^1^H NMR ([D_6_]DMSO, 500 MHz, 25 °C, TMS): *δ* = 1.12 (d, 12H), 1.17 (d, 6H), 2.81 (t, 1H), 4.53 (t, 2H), 6.98 (s, 2H), 9.59 (s, 2H) ppm. ^13^C NMR ([D_6_]DMSO, 125 MHz, 25 °C): *δ* = 24.78, 25.26, 28.59, 33.75, 66.83, 121.99, 141.57, 147.37, 148.12 ppm. IR (KBr pellet): *ν* = 3333, 3265, 3187, 2960, 2870, 1637, 1599, 1461, 1378, 1318, 1182, 1086, 1014, 881 cm^−1^. Anal. calcd for C_15_H_25_NO_3_S: C 60.17, H 8.42, N 4.68%; found C 60.37, H 8.35, N 4.73%.

#### Compound PHA

Yellow solid, with a yield of 82.9%. ^1^H NMR ([D_6_]DMSO, 500 MHz, 25 °C, TMS): *δ* = 5.00 (s, 2H), 9.01 (s, 1H), 9.04 (s, 1H) ppm. ^13^C NMR ([D_6_]DMSO, 125 MHz, 25 °C): *δ* = 124.08, 125.39 ppm. IR (KBr pellet): *ν* = 3331, 3303, 3266, 3084, 1552, 1540, 1360, 1341 cm^−1^. Anal. calcd for C_6_H_6_N_4_O_7_: C 29.52, H 1.65, N 22.95%; found C 29.43, H 1.72, N 22.88%. MS (*m*/*z*): 243.06 [M − H]^−^.

### 1-Amino-3,5-dinitro-1,2,4-triazole (ADNT)

3,5-Dinitro-1,2,4-triazole (DNT) (2.0 g, 0.13 mol) was dissolved in ethanol (15.0 mL) at room temperature, then potassium hydroxide (5%, 14.2 g, 0.13 mol) was added dropwise at 5–10 °C. After 1 h at room temperature, the precipitate was filtered off, washed with ethanol and ice-water, and dried in air to give yellow solid potassium 3,5-dinitro-1,2,4-triazolate (KDNT). KDNT (1.0 g, 5.1 mmol) was dispersed in anhydrous acetonitrile (40.0 mL) at room temperature. A solution of MSH (1.62 g, 7.5 mmol) and acetonitrile (10.0 mL) was added dropwise for 0.5 h. After complete addition, the mixture was stirred at 20–25 °C for another 10 h, evaporated organic solvent under reduced pressure, recrystallized from acetone, dried in air to obtain 0.73 g yellow solid ADNT with a yield of 82.6%. ^1^H NMR ([D_6_]DMSO, 500 MHz, 25 °C, TMS): *δ* = 6.83 (s, 2H) ppm. ^13^C NMR ([D_6_]DMSO, 125 MHz, 25 °C): *δ* = 155.31, 155.92 ppm. IR (KBr pellet): *ν* = 3424, 3340, 3297, 3229, 1671, 1520, 1313 cm^−1^. Anal. calcd for C_2_H_2_N_6_O_4_: C 13.80, N 48.28, H 1.16%; found C 13.96, N 48.01, H 1.22%. MS (*m*/*z*): 174.05 [M^+^].

### 1,1′-Azobis(3,5-dinitro-1,2,4-triazole) (ABDNT)

ADNT (0.1 g, 0.58 mmol) was dissolved in anhydrous acetonitrile (10.0 mL) under the existence of nitrogen gas at room temperature, then *tert*-butyl hypochlorite (*t*-BuOCl) (0.082 g, 0.75 mmol) was slowly added dropwise at 0–5 °C. After complete addition, the mixture was stirred at 20–25 °C for another 20 h, evaporated acetonitrile under reduced pressure, and triturated with isopropyl alcohol (1.5 mL). The precipitate was filtered off, washed with isopropyl alcohol and ice-water, and dried to give 41.0 mg yellow solid ABDNT with a yield of 41.5%. ^13^C NMR ([D_6_]DMSO, 125 MHz, 25 °C): *δ* = 133.45, 141.66 ppm. IR (KBr pellet): *ν* = 1661, 1657, 1570, 1371, 1329, 1397, 1250, 1122, 1012 cm^−1^. Anal. calcd for C_4_N_12_O_8_: C 13.96, N 48.84%; found C 13.87, N 48.91%. MS (*m*/*z*): 344.06 [M^+^].

### 1,1′-Azobis(3-chloro-5-nitro-1,2,4-triazole) (ABCNT)

ADNT (0.174 g, 1.0 mmol) was dissolved in anhydrous acetonitrile (15.0 mL) under the existence of nitrogen gas at room temperature, then *tert*-butyl hypochlorite (*t*-BuOCl) (0.273 g, 2.5 mmol) was slowly added dropwise at 0–5 °C. After complete addition, the mixture was stirred at 20–25 °C for another 20 h, then ethyl acetate (30.0 mL) was added, washed organic phase with ice-water, dried with magnesium sulfate, evaporated organic solvent under reduced pressure and dried in air to obtain 0.113 g light-yellow solid ABCNT with a yield of 65.7%. ^13^C NMR ([D_6_]DMSO, 125 MHz, 25 °C): *δ* = 141.73, 158.73 ppm. IR (KBr pellet): *ν* = 1650, 1574, 1507, 1446, 1388, 1347, 1303, 1242, 1218, 1097, 1000, 844, 743 cm^−1^. Anal. calcd for C_4_N_10_O_4_Cl_2_: C 14.87, N 43.36%; found C 14.93, N 43.29%.

### 1,1′-Azobis(3,5-diazido-1,2,4-triazole) (ABDAT)

ADNT (0.174 g, 1.0 mmol) was dissolved in anhydrous acetonitrile (15.0 mL) under the existence of nitrogen gas at room temperature, then *tert*-butyl hypochlorite (*t*-BuOCl) (0.273 g, 2.5 mmol) was slowly added dropwise at 0–5 °C. After complete addition, the mixture was stirred at 20–25 °C for another 20 h, then ethyl acetate (30.0 mL) was added, washed organic phase with ice-water, dried with magnesium sulfate, evaporated organic solvent under reduced pressure and dried in air to obtain 0.113 g light-yellow solid ABCNT with a yield of 65.7%. ^13^C NMR ([D_6_]DMSO, 125 MHz, 25 °C): *δ* = 151.65, 157.41 ppm. ^14^N NMR ([D_6_]DMSO, 60 MHz, 25 °C): *δ* = −275.12, −229.05, −145.99, −145.17, −142.52, −130.17, −68.91, −1.382 ppm. IR (KBr pellet): *ν* = 2405, 2288, 2208, 2182, 2155, 1542, 1515, 1406, 1343, 1291, 1212, 1200, 1086, 999 cm^−1^. Anal. calcd for C_4_N_20_: C 14.64, N 85.36%; found C 14.76, N 85.21%. MS (*m*/*z*): 351.10 [M + Na]^+^.

## Conclusions

Three novel catenated N_6_ energetic derivatives based on 1-amino-3,5-dinitro-1,2,4-triazole (ADNT), which contain 1,1′-azobis(3,5-dinitro-1,2,4-triazole) (ABDNT), 1,1′-azobis(3-chloro-5-nitro-1,2,4-triazole) (ABCNT) and 1,1′-azobis(3,5-diazido-1,2,4-triazole) (ABDAT), were prepared and fully characterized by using IR and NMR spectroscopies, elemental analysis, mass spectra, and single-crystal X-ray diffraction analysis for ABCNT. These energetic derivatives exhibit acceptable thermal stabilities (168.8–262.4 °C). Densities for these compounds fall in the range between 1.71 and 1.93 g cm^−3^. All of the compounds exhibit higher positive heats of formation ranging between 774.8 and 2150.8 kJ mol^−3^. Especially, compound ABDAT exhibits the highest heat of formation (2150.8 kJ mol^−1^), which is higher than other reported catenated nitrogen-atom compounds. Their detonation properties were evaluated by Gaussian 09 and Kamlet–Jacobs equations. The calculated detonation velocities (8.22–9.49 km s^−1^) and detonation pressures (29.6–42.4 GPa) are comparable to those of explosives such as TNT (6.88 km s^−1^, 19.5 GPa) and RDX (8.71 km s^−1^, 33.7 GPa). Most of these compounds have reasonable impact sensitivities (6 to 20 J) and friction sensitivities (80 to 300 N), which suggest that they have the potential to be useful new energetic materials.

## Conflicts of interest

There are no conflicts to declare.

## Supplementary Material

RA-008-C8RA02491J-s001
