# Supplementary material for: Novel catenated N6 energetic compounds based on substituted 1,2,4-triazoles: synthesis, structures and properties
Source: RSC Adv. 2018 Apr 16;8(25):13755–63. doi: 10.1039/c8ra02491j (PMC9079829; doi:10.1039/c8ra02491j)
Supplement: RA-008-C8RA02491J-s001 [file RA-008-C8RA02491J-s001.pdf]

Electronic Supplementary Material (ESI) for RSC Advances.
This journal is © The Royal Society of Chemistry 2018

Supporting Information

Novel catenated N_6 energetic compounds based on substituted 1,2,4-triazoles: syntheses, structures and properties

Yanan Li,*[a] Bin Wang,[a] Pei Chang,[a] Jianjian Hu,[a] Tao Chen,[a] Yinglei Wang,*[a] and Bozhou Wang[a]

State Key Laboratory of Fluorine & Nitrogen Chemicals, Xi'an Modern Chemistry Research Institute, Xi'an, Shanxi 710065, People's Republic of China

Email: lyn2003080094@126.com

Table of Contents

1. X-ray crystallography

2. NMR spectroscopies and DSC curves

3. Theoretical study

1. X-ray crystallography

Single crystals suitable for X-ray measurement was obtained by slow evaporation of aqueous solution of ABCNT at room temperature. Bond lengths, bond angles and torsion angles of the data collection and refinement are given in Table S1–S2. The CIF files of ABCNT have been deposited at the Cambridge Crystallographic Data Centre as supplementary publication 1826501.

Table S1. Bond lengths[Å] for the structure of ABCNT.

Cl(1)-C(1)	1.672(5)	N(6)-N(6)#2	1.248(8)
Cl(2)-C(3)	1.670(6)	N(6)-N(7)	1.384(5)
N(1)-N(1)#1	1.240(7)	N(7)-N(8)	1.348(6)
N(1)-N(2)	1.373(5)	N(7)-C(3)	1.361(6)
N(2)-C(1)	1.352(6)	N(8)-C(4)	1.310(6)
N(2)-N(3)	1.369(5)	N(9)-C(3)	1.306(7)
N(3)-C(2)	1.291(6)	N(9)-C(4)	1.349(7)
N(4)-C(1)	1.306(6)	N(10)-O(4)	1.212(6)
N(4)-C(2)	1.340(6)	N(10)-O(3)	1.213(6)
N(5)-O(1)	1.192(6)	N(10)-C(4)	1.454(7)
N(5)-O(2)	1.209(6)		
N(5)-C(2)	1.459(7)		

Symmetry transformations used to generate equivalent atoms: #1 -x,-y,-z; #2 -x+1,-y,-z+2

Table S2. Bond angles [deg] andtorsion angles [deg] for the structure of ABCNT.

N(1)#1-N(1)-N(2)	110.7(5)	N(1)#1-N(1)-N(2)-C(1)	-175.0(5)
C(1)-N(2)-N(3)	109.4(4)	N(1)#1-N(1)-N(2)-N(3)	8.2(8)
C(1)-N(2)-N(1)	124.0(4)	C(1)-N(2)-N(3)-C(2)	-0.5(5)
N(3)-N(2)-N(1)	126.5(4)	N(1)-N(2)-N(3)-C(2)	176.8(4)
C(2)-N(3)-N(2)	99.8(4)	N(6)#2-N(6)-N(7)-N(8)	2.7(8)
C(1)-N(4)-C(2)	100.7(4)	N(6)#2-N(6)-N(7)-C(3)	-176.9(5)
O(1)-N(5)-O(2)	124.9(5)	C(3)-N(7)-N(8)-C(4)	-0.2(6)
O(1)-N(5)-C(2)	117.3(5)	N(6)-N(7)-N(8)-C(4)	-179.8(4)
O(2)-N(5)-C(2)	117.7(5)	C(2)-N(4)-C(1)-N(2)	0.8(6)
N(6)#2-N(6)-N(7)	109.0(5)	C(2)-N(4)-C(1)-Cl(1)	-179.6(4)
N(8)-N(7)-C(3)	110.5(4)	N(3)-N(2)-C(1)-N(4)	-0.2(6)
N(8)-N(7)-N(6)	126.6(4)	N(1)-N(2)-C(1)-N(4)	-177.6(4)
C(3)-N(7)-N(6)	122.9(4)	N(3)-N(2)-C(1)-Cl(1)	-179.9(3)
C(4)-N(8)-N(7)	100.2(4)	N(1)-N(2)-C(1)-Cl(1)	2.8(7)
C(3)-N(9)-C(4)	101.6(5)	N(2)-N(3)-C(2)-N(4)	1.1(6)
O(4)-N(10)-O(3)	125.7(5)	N(2)-N(3)-C(2)-N(5)	-179.0(4)
O(4)-N(10)-C(4)	117.3(5)	C(1)-N(4)-C(2)-N(3)	-1.2(6)
O(3)-N(10)-C(4)	117.0(6)	C(1)-N(4)-C(2)-N(5)	178.8(5)
N(4)-C(1)-N(2)	110.7(4)	O(1)-N(5)-C(2)-N(3)	9.0(7)
N(4)-C(1)-Cl(1)	126.5(4)	O(2)-N(5)-C(2)-N(3)	-168.6(5)

N(2)-C(1)-Cl(1)	122.8(4)	O(1)-N(5)-C(2)-N(4)	-171.0(5)
N(3)-C(2)-N(4)	119.3(5)	O(2)-N(5)-C(2)-N(4)	11.3(8)
N(3)-C(2)-N(5)	119.5(5)	C(4)-N(9)-C(3)-N(7)	-1.5(6)
N(4)-C(2)-N(5)	121.2(5)	C(4)-N(9)-C(3)-Cl(2)	-179.9(5)
N(9)-C(3)-N(7)	109.8(5)	N(8)-N(7)-C(3)-N(9)	1.2(7)
N(9)-C(3)-Cl(2)	127.0(4)	N(6)-N(7)-C(3)-N(9)	-179.1(4)
N(7)-C(3)-Cl(2)	123.2(4)	N(8)-N(7)-C(3)-Cl(2)	179.6(4)
N(8)-C(4)-N(9)	117.8(5)	N(6)-N(7)-C(3)-Cl(2)	-0.8(8)
N(8)-C(4)-N(10)	119.9(5)	N(7)-N(8)-C(4)-N(9)	-0.9(6)
N(9)-C(4)-N(10)	122.3(5)	N(7)-N(8)-C(4)-N(10)	-179.8(5)
		C(3)-N(9)-C(4)-N(8)	1.6(7)
		C(3)-N(9)-C(4)-N(10)	-179.6(5)
		O(4)-N(10)-C(4)-N(8)	13.9(8)
		O(3)-N(10)-C(4)-N(8)	-164.6(5)
		O(4)-N(10)-C(4)-N(9)	-165.0(5)
		O(3)-N(10)-C(4)-N(9)	16.6(8)

Symmetry transformations used to generate equivalent atoms: #1 -x,-y,-z; #2 -x+1,-y,-z+2

2. NMR spectra of all compounds

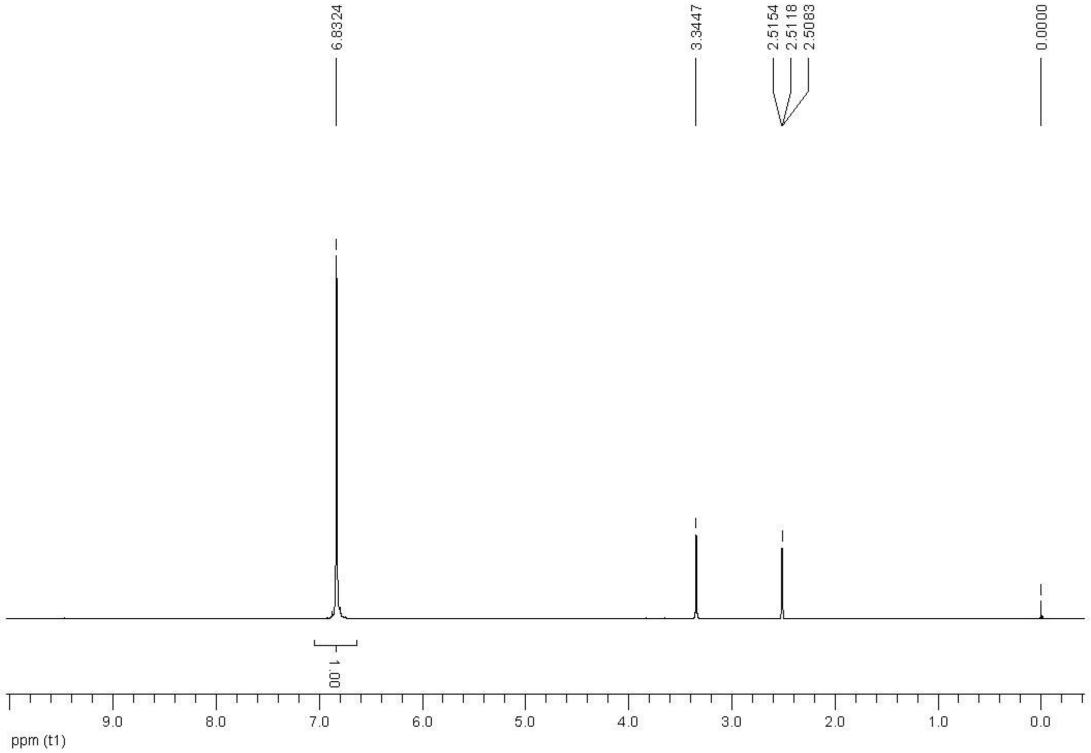

Fig. S1 ^{1}H NMR spectrum of ADNT in DMSO-d_6

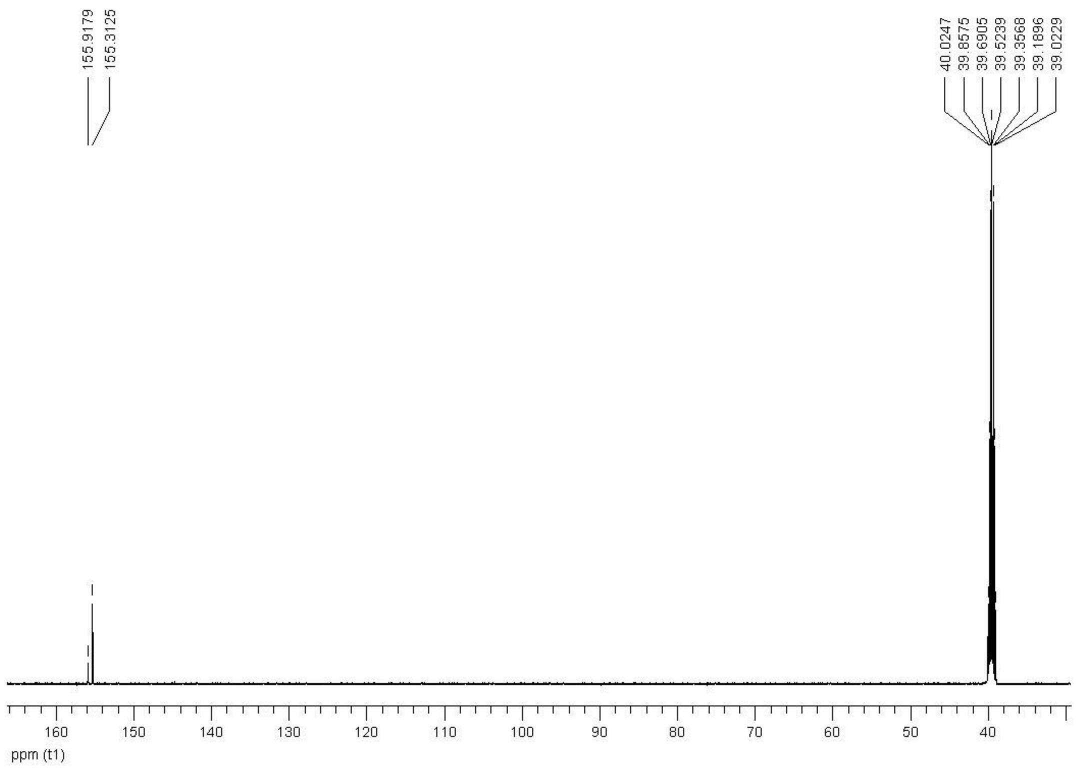

Fig. S2 ^{13}C NMR spectrum of ADNT in DMSO-d_6

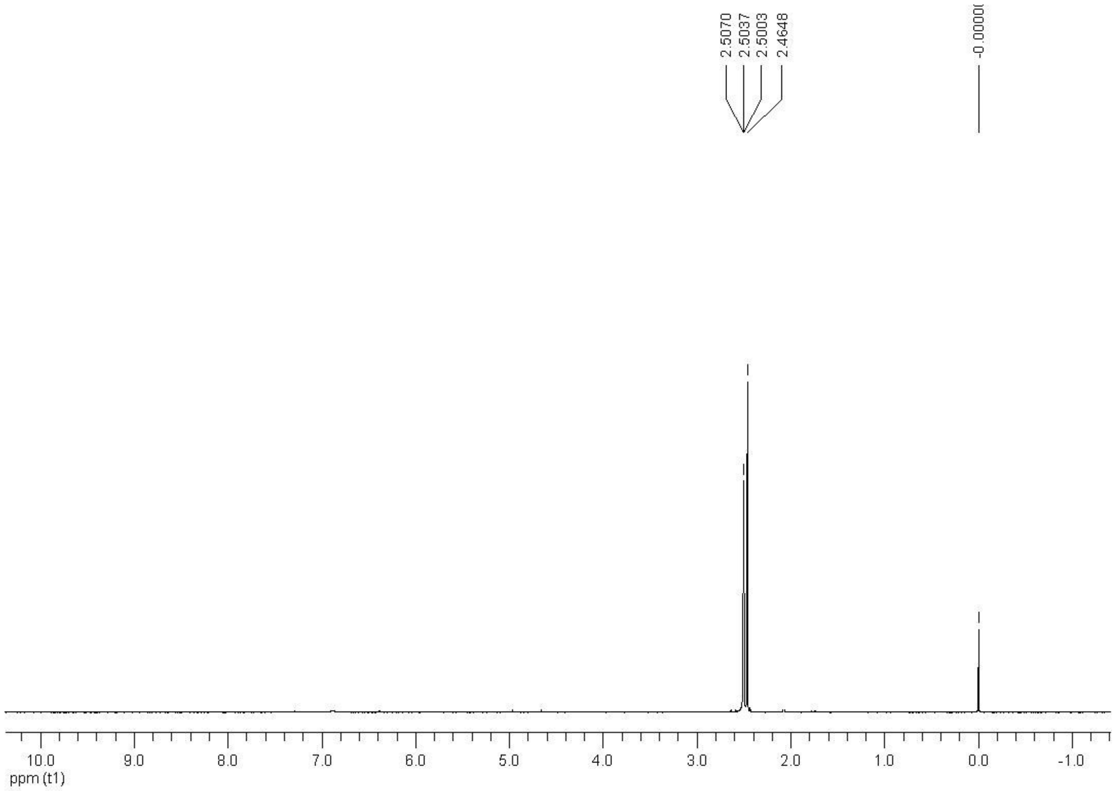

Fig. S3 ^{1}H NMR spectrum of ABDNT in DMSO-d_6

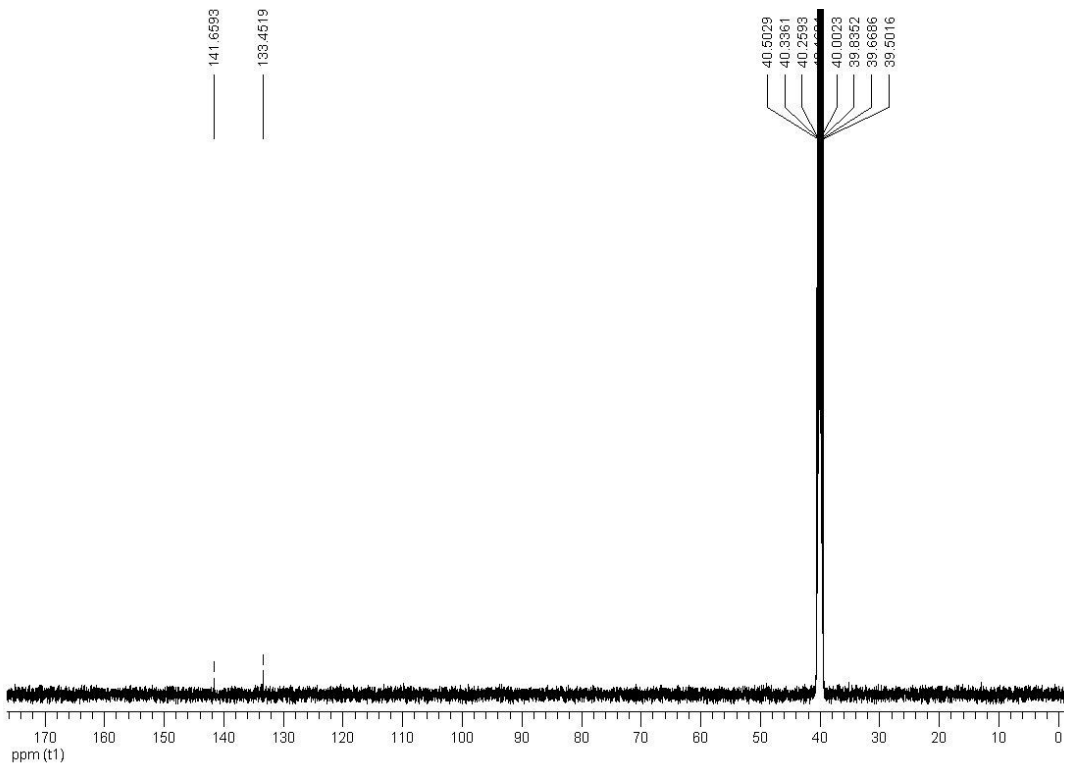

Fig. S4 ^{13}C NMR spectrum of ABDNT in DMSO-d_6

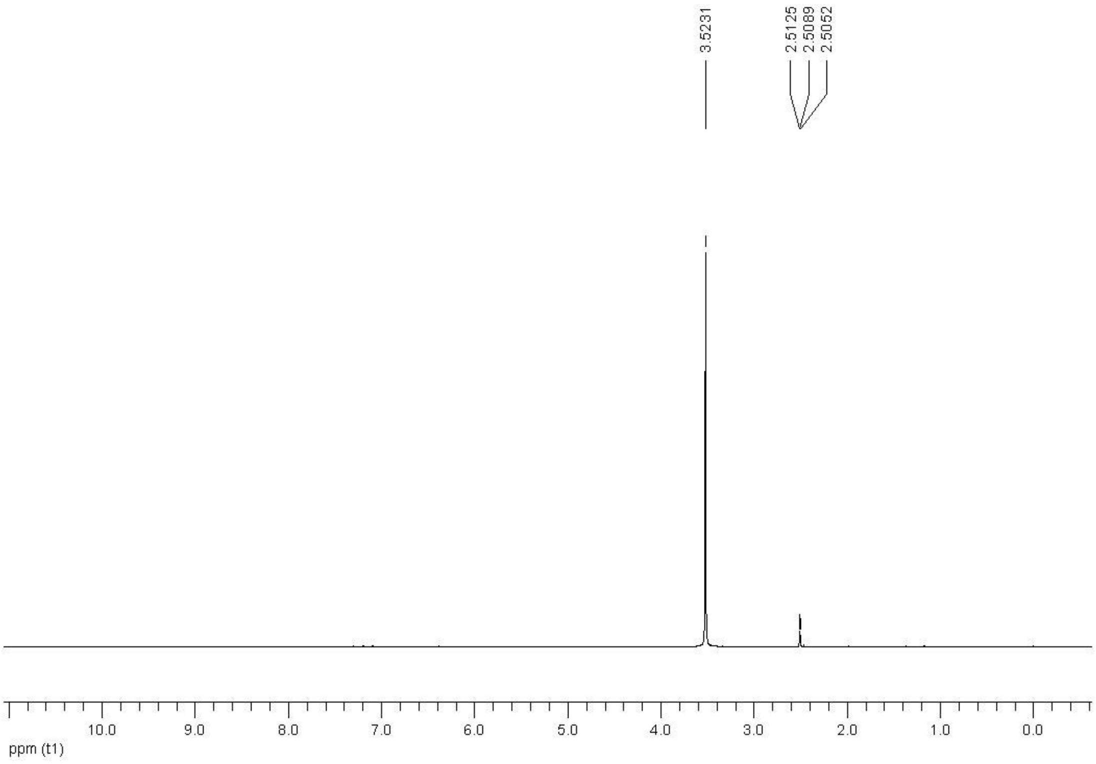

Fig. S5 ^{1}H NMR spectrum of ABCNT in DMSO-d_6

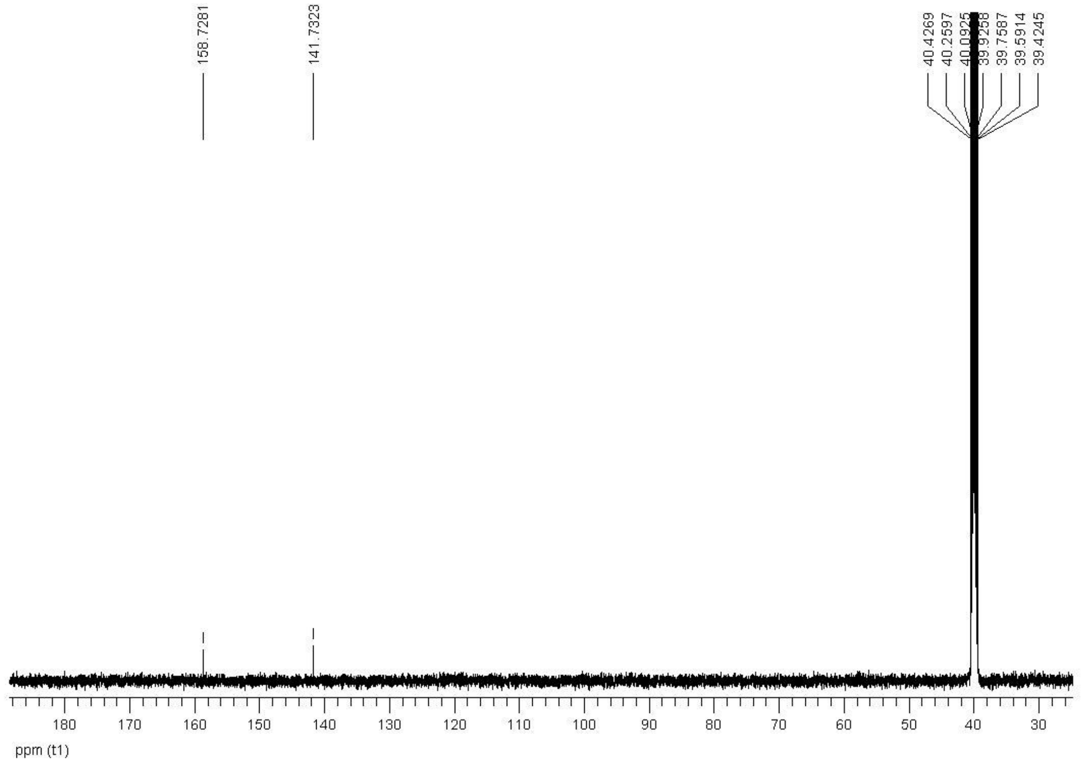

Fig. S6 ^{13}C NMR spectrum of ABCNT in DMSO-d_6

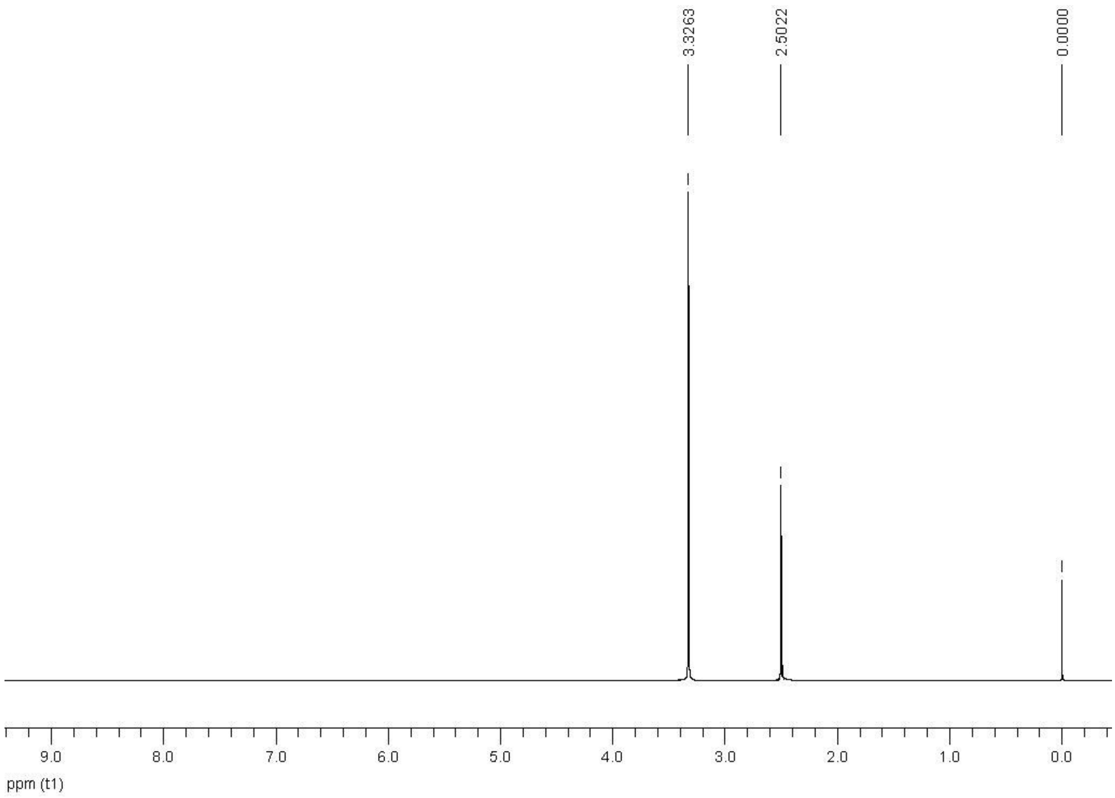

Fig. S7 ^{1}H NMR spectrum of ABDAT in DMSO-d_6

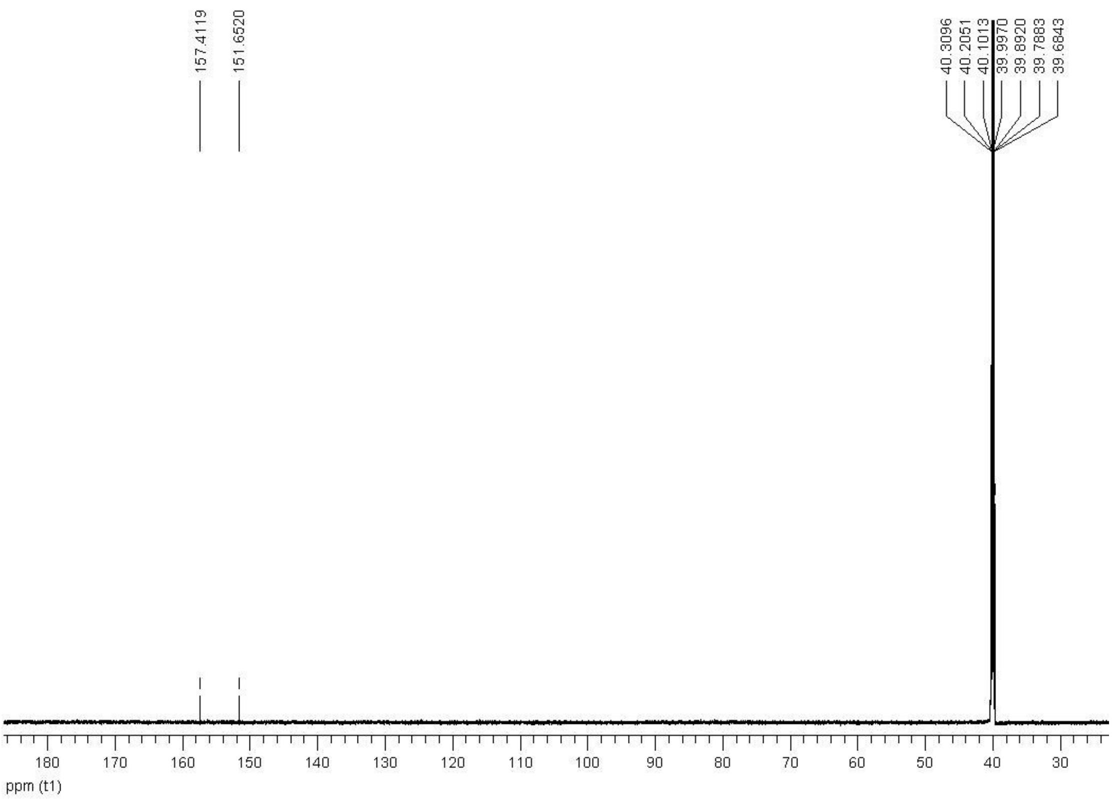

Fig. S8 ^{13}C NMR spectrum of ABDAT in DMSO-d_6

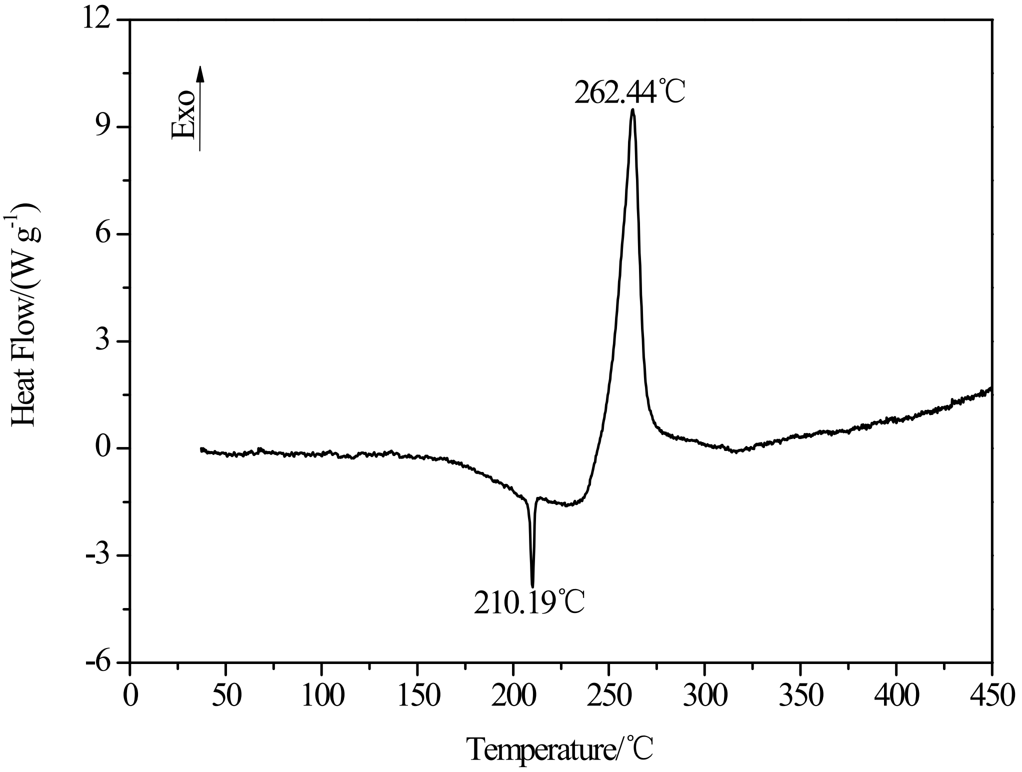

Fig. S9 DSC curve of ABDNT at heating rate of 5℃ min^{-1}

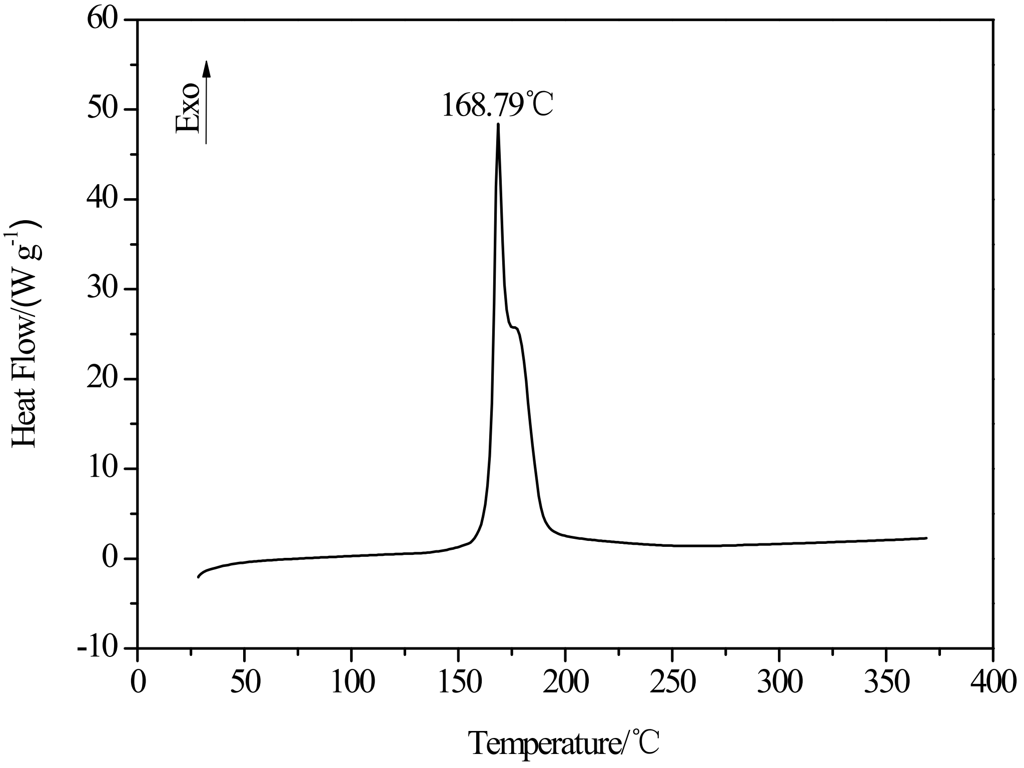

Fig. S10 DSC curve of ABDAT at heating rate of 5°C min^{-1}

3. Theoretical study

All quantum chemical calculations were carried out by using the Gaussian 09 (revision A.02) program package and visualized by Gauss View 5.05.[1,2] The gas phase enthalpies of formation (Table S3) were obtained by the atomization method (equation (1)) based on CBS-4M calculated electronic enthalpies using NIST[3] values as standardized values for the standard heats of formation ($\Delta_f H°$) (Table S4).

$$\Delta_f H°_{(g, M, 298)} = H_{(Molecule, 298)} - \sum H°_{(Atoms, 298)} + \sum \Delta_f H°_{(Atoms, 298)} \quad (1)$$

The solid state enthalpies of formation (Table S3) can be determined using the gas-phase enthalpy of formation and enthalpy of sublimation phase transition according to the Hess' law of constant heat summation as the equation (2).[4]

$$\Delta H(\text{solid}) = \Delta H(\text{gas}) - \Delta H(\text{sublimation}) \quad (2)$$

Based on the electrostatic potential of a molecule by quantum mechanical prediction, the heat of sublimation can be represented as the equation (3).[5]

$$\Delta H(sub\lim ation) = a(SA)^2 + b(\sigma_{Tot}{}^2 \upsilon)^{1/2} + c \quad (3)$$

Table S3. Heats of formation based on CBS-4M calculations

Compound	$-H_{(M, 298)}$[a] / a.u.	$\Delta_f H°_{(g)}$[b] / kJ mol^{-1}	SA[c] / Å^2	$\sigma_{Tot}{}^2\, \upsilon$[d] / (kcal mol^{-1})^2	$\Delta H°_{(sub)}$[e] / kJ mol^{-1}	$\Delta_f H°_{(s)}$[f] / kJ mol^{-1}
**ABDNT**	1408.902440	1192.06	306.93	20.70	218.156	973.90

**ABCNT**	1918.711869	901.34	285.05	29.38	126.535	774.80
**ABDAT**	1245.373322	2399.75	328.68	24.21	248.921	2150.83

[a] CBS-4M electronic enthalpy; [b] gas phase enthalpy of formation; [c] surface area; [d] electrostatic potential of the molecular surface; [e] heat of sublimation; [f] standard solid state heat of formation.

Table S4. CBS-4M values and literature values for atomic $\Delta_f H°$/ kJ mol^{-1}

	$-H^{298}$ / a.u.	NIST[5]/ $\Delta_f H^{\circ}_{(298K)}$
H	0.500991	218.2
C	37.786156	717.2
N	54.522462	473.1
O	74.991202	249.5
Cl	459.676936	121.3

References:

[1] M. J. Frisch, G. W. Trucks, H. B. Schlegel, G. E. Scuseria,M. A. Robb, J. R. Cheeseman, J. A. Montgomery Jr, T. Vreven, K. N. Kudin, J. C. Burant, J. M. Millam, S. S. Iyengar, J. Tomasi, V. Barone, B. Mennucci, M. Cossi, G. Scalmani, N. Rega, G. A. Petersson, H. Nakatsuji, M. Hada, M. Ehara, K. Toyota, R. Fukuda, J. Hasegawa, M. Ishida, T. Nakajima, Y. Honda, O. Kitao, H. Nakai, M. Klene, X. Li, J. E. Knox, H. P. Hratchian, J. B. Cross, V. Bakken, C. Adamo, J. Jaramillo, R. Gomperts, R. E. Stratmann, O. Yazyev, A. J. Austin, R. Cammi, C. Pomelli, J. W. Ochterski, P. Y. Ayala, K. Morokuma, G. A. Voth, P. Sal-vador, J. J. Dannenberg, V. G. Zakrzewski, S. Dapprich, A. D. Daniels, M. C. Strain, O. Farkas, D. K. Malick, A. D. Rabuck, K. Raghavachari, J. B. Foresman, J. V. Ortiz, Q. Cui, A. G. Ba-boul, S. Clifford, J. Cioslowski, B. B. Stefanov, G. Liu, A. Liashenko, P. Piskorz, I. Komaromi, R. L. Martin, D. J. Fox, T. Keith, M. A. Al-Laham, C. Y. Peng, A. Nanayakkara, M. Challacombe, P. M. W. Gill, B. Johnson, W. Chen, M. W. Wong, C. Gonzalez and J. A. Pople, Gaussian 09, rev. A.02, Gaussian, Inc., Wallingford, CT, 2009.

[2] T. K. R. Dennington and J. Millam, GaussView 5, V5.0.8,Semichem Inc., Shawnee Mission, 2009.

[3] P. J. Lindstrom, W. G. Mallard, NIST Chemistry Webbook,NIST Standard Reference 69, June 2005, National Instituteof Standards and Technology, Gaithersburg, MD(http://webbook.nist.gov).

[4] P. W. Atkins, Physical Chemistry, Oxford University Press, Oxford, 1982.

[5] P. Politzer, J. S. Murray, T. Brinck and P. Lane, in Immunoanalysis of Agrochemicals, ACS Symp.Ser. 586, American Chemical Society, Washington, DC, 1994.
